# Ankylosing spondylitis and psychiatric disorders in European population: a Mendelian randomization study

**DOI:** 10.3389/fimmu.2023.1277959

**Published:** 2023-10-26

**Authors:** Huifang Zuo, Min-Min Li

**Affiliations:** Department of Clinical Laboratory Medicine, The First Affiliated Hospital of Jinan University, Guangzhou, China

**Keywords:** ankylosing spondylitis, psychiatric disorders, Mendelian randomization, major depression, anxiety disorder, schizophrenia, bipolar disorder, anorexia nervosa

## Abstract

**Background:**

Epidemiologic evidence has demonstrated a correlation between ankylosing spondylitis and psychiatric disorders. However, little is known about the common genetics and causality of this association. This study aimed to investigate the common genetics and causality between ankylosing spondylitis (AS) and psychiatric disorders.

**Methods:**

A two-sample Mendelian Randomization (MR) analysis was carried out to confirm causal relationships between ankylosing spondylitis and five mental health conditions including major depressive disorder (MDD), anxiety disorder (AXD), schizophrenia (SCZ), bipolar disorder (BIP), and anorexia nervosa (AN). Genetic instrumental variables associated with exposures and outcomes were derived from the largest available summary statistics of genome-wide association studies (GWAS). Bidirectional causal estimation of MR was primarily obtained using the inverse variance weighting (IVW) method. Other MR methods include MR-Egger regression, Weighted Median Estimator (WME), Weighted Mode, Simple Mode, and Mendelian randomization pleiotropy residual sum and outlier (MR-PRESSO). Sensitivity analyses are conducted to estimate the robustness of MR results.

**Results:**

The findings suggest that AS may be causally responsible for the risk of developing SCZ (OR = 1.18, 95% confidence interval = (1.06, 1.31), P = 2.58 × 10^-3^) and AN (OR = 1.32, 95% confidence interval = (1.07, 1.64), P = 9.43 × 10^-3^). In addition, MDD, AXD, SCZ, AN, and BIP were not inversely causally related to AS (all p > 0.05).

**Conclusion:**

Our study provides fresh insights into the relationship between AS and psychiatric disorders (SCZ and AN). Furthermore, it may provide new clues for risk management and preventive interventions for mental disorders in patients with AS.

## 1 Introduction

Ankylosing spondylitis (AS) is a chronic progressive inflammatory disease with the axial skeleton as the main site of development, leading to typically inflammatory low back pain, as well as severe structural and functional deficits (1). The prevalence of the disease is reported to be between 0.07% and 0.32% worldwide and is 2-3 times more prevalent in men than in women (2). The etiology of AS has not been elucidated. Genetics, infection, gut microbiota, mechanical stress, gender, and environmental and lifestyle factors have been identified as risk factors for AS (3).

A study on the worldwide burden of diseases showed that the burden caused by psychiatric disorders is worsening (4). Psychiatric disorders are one of the major causes of death all over the world (5). Notably, in addition to chronic pain and disability, people with AS often suffer from psychiatric disorders, with depression and anxiety being the most common psychological traits (6, 7). A previous study showed that patients with AS had an increased risk of affective disorders (depression and bipolar disorder [BIP]) and anxiety disorder (AXD), while the prevalence of schizophrenia (SCZ) was not above that of the general population (8). Another retrospective study found that AS was not associated with subsequent new diagnoses of BIP and SCZ (9). Conversely, Kang et al. reported a significantly increased prevalence of comorbid psychoses in individuals with AS (10). Moreover, a population-based cross-sectional study suggests that AS reduces the risk of SCZ (11). Evidently, these clinical observational studies have yielded conflicting outcomes. Furthermore, it is worth noting that an earlier diagnosis of AS or psychiatric disorders does not invariably signify an earlier onset in many cases. Additionally, it is imperative to acknowledge that observational studies may encounter potential confounding factors such as reverse causation and selection bias, which can impact their findings. Consequently, the precise relationship between AS and psychiatric disorders remains elusive.

Since alleles are randomly assigned as they are inherited from parents to offspring, the process can be considered analogous to randomized grouping in a randomized controlled trial. Moreover, genotypes are innately determined and are not interfered with by confounding factors such as growth environment, socioeconomic status, and behavioral traits. In the Mendelian randomization (MR) investigation, genetic variants closely associated with risk factors were utilized as instrumental variables (IVs) to provide evidence of a causal relationship between exposure and outcome (12). This approach is being increasingly adopted because it addresses the primary limitation of evidence from observational studies: the lack of measurement of confounding factors (13). In the present study, two-sample bidirectional MR and multivariate MR analyses were conducted utilizing genome-wide association studies (GWAS) statistics to investigate the potential role of AS as a risk factor for psychiatric disorders and the possibility of reverse causality between psychiatric disorders and AS.

## 2 Materials and methods

### 2.1 Mendelian randomization study

**Figure 1:**
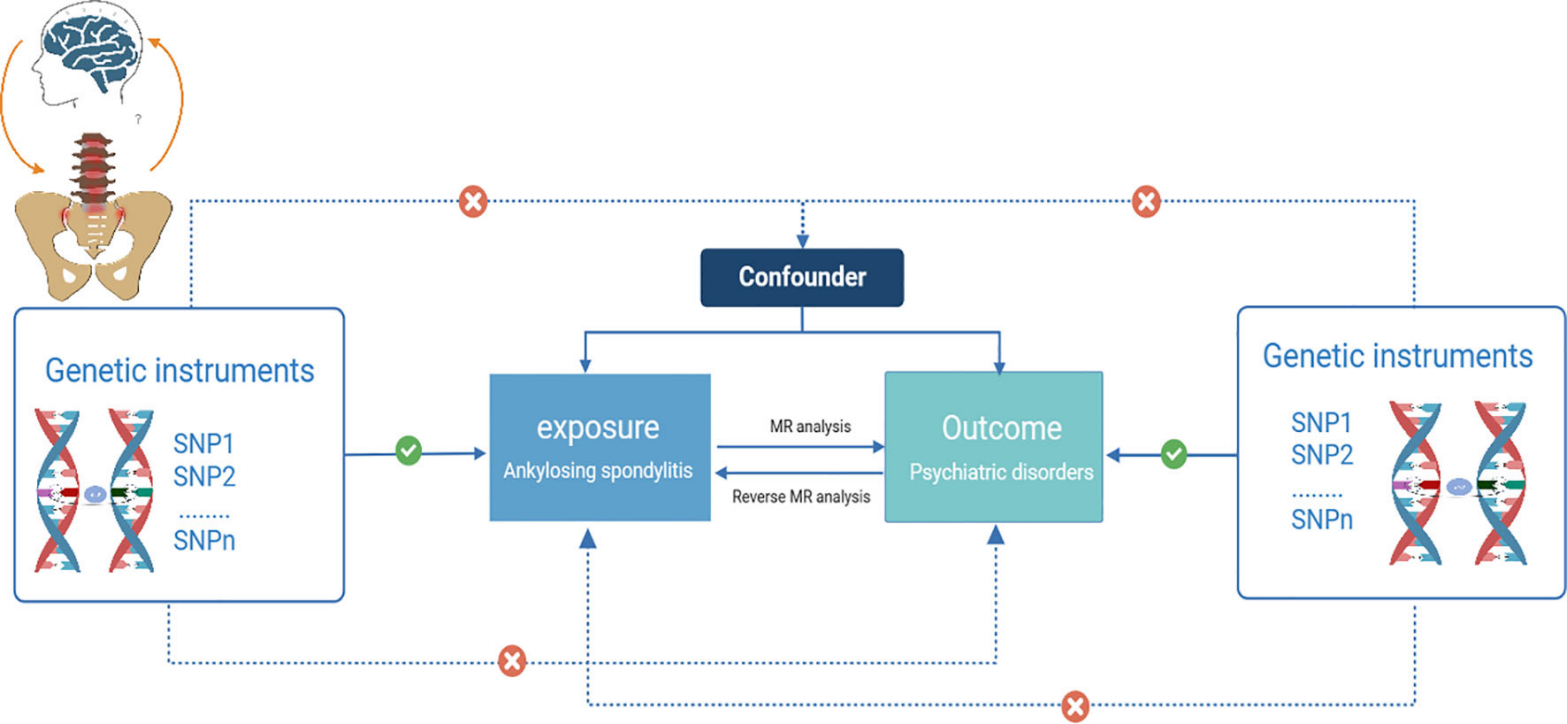
Schematic diagram of a two-sample Mendelian randomization study (Visualization of causal relationships between genetic instrumental variables, exposures, and outcomes. Solid arrows indicate permissible relationships between variables under the assumptions of Mendelian randomization, and dashed lines indicate prohibited relationships for valid IVs).

Three core hypotheses for MR studies should be met (14): (i) Genetic IVs are robustly and strongly associated with AS (association hypothesis); (ii) Genetic IVs are independent of confounders influencing the exposure-outcome relationship (independence hypothesis); and (iii) Genetic IVs affect psychiatric disorders exclusively through AS (exclusivity hypothesis) (15). We examined potential pleiotropy using a range of MR methods to validate the second and third MR hypotheses. Horizontal pleiotropy exists if the variants affect the target outcome through other traits than the exposure pathway, or if the variants have a direct effect on the outcome (16). Similar hypotheses were applied to reverse MR analysis. A schematic diagram of the MR causality study design is shown in **Figure 1**, which explains the basic principles of MR studies.

### 2.2 Sources of statistical data

GWAS summary data is an umbrella term for a series of data that respond to the effect of single nucleotide polymorphisms (SNPs) on phenotype. GWAS data associated with AS were collected from the IGAS (International Genetics of Ankylosing Spondylitis) Consortium comprising 22,647 subjects of European ancestry (9,069 cases and 13,578 controls) (17). Diagnostic criteria for AS are based on the modified New York (mNY) criteria (18). Genetic data associated with the four psychiatric disorders MDD, BIP, SCZ, and AN were sourced from the Psychiatric Genomics Consortium (PGC) database (https://www.med.unc.edu/pgc/download-results) to data overlap to ensure independence between two samples. Additionally, we have extracted GWAS summary statistics for AXD from the FinnGen consortium as part of our analysis(https://www.finngen.fi/fi) (19). MDD GWAS summary statistics were gathered from the largest GWAS meta-analysis of depression, which included 246,363 cases and 561,190 controls. It is worth mentioning, however, that the GWAS summary statistics provided by the 23andMe company have not yet been made accessible to the public. Consequently, our present MR analysis relied on a cohort comprising 170,756 individuals diagnosed with MDD and 329,443 control subjects (20). Details of the GWAS data are shown in **Supplementary Table S1**. Case definitions for AS and the 5 mental disorders are in **Supplementary Table S2**.

In our current MR study, to minimize potential bias due to population heterogeneity, individuals with AS or psychiatric disorders were restricted to European ancestry (21). No additional ethical approvals were required as data from public databases allowed open access to researchers.

### 2.3 Selection of IVs

Effective IVs were identified through careful filtering of the GWAS data, adhering to the core assumptions of MR. Initially, we searched for significant SNPs that were strongly correlated with AS at a genome-wide significance threshold of p-value < 5 × 10-8. Subsequently, SNPs with linkage disequilibrium (LD) were removed to ensure independence between IVs (r2 = 0.001, KB = 10,000), as LD signifies a non-random association between alleles. Furthermore, we excluded allele frequencies that were less than 0.01 in terms of minor allele frequency (MAF). In cases where an exposure SNP was absent in the outcome-related GWAS summary data, it was substituted with a highly correlated SNP (r^2^ > 0.8). IVs lacking alternative SNPs as well as outcome-related IVs (p < 0.05) were excluded from the analysis. After that, we employed harmonized effects to ensure effect allele alignments for exposure and outcome IVs, eliminating palindromic SNPs (defined as SNPs with A/T or G/C alleles) with moderate allele frequencies (MAF > 0.42). Finally, we estimated the strength of the association between SNPs and exposure using the F-statistic, calculated as follows: F = β^2^/SD^2^, where β denotes the effect size of SNPs on exposure and SD denotes the standard deviation (22). To avoid bias in effect estimation, genetic variants with an F value below 10 were excluded, indicating their weak ability to explain exposure (23). The effective SNPs for the estimation of the causal effect of AS on psychiatric disorders, which were derived after a rigorous screening process, are presented in **Supplementary Table S3**.

### 2.4 Statistical methods

As the primary and most accurate causal effect estimate, the inverse variance weighted (IVW) approach presumes that all SNPs are valid IVs. It estimates the overall causal effect by calculating the Wald Ratio (WR), which is the regression coefficient of the IV on the outcome divided by the regression coefficient of the IV on the exposure, for each SNP (15). The multiplicative random-effects IVW approach yields more conserved causal inferences than fixed-effects IVW and takes into account the uncertainty associated with pleiotropy. All “IVW” in this study refers to random-effects IVW unless otherwise stated (24). The IVW method yields robust findings when all IVs exhibit validity and there is no presence of horizontal pleiotropy. For genetic instruments involving a single SNP, we employed the WR. Additionally, other methods including MR-Egger regression, Weighted Median Estimator (WME), Weighted Mode, Simple Mode, and Mendelian randomization pleiotropy residual sum and outlier (MR-PRESSO) were employed to supplement the results of MR to evaluate reliability and stability. The MR-Egger method, which considers that all genetic variants are null IVs, provides a valid test of the null causality hypothesis (14). This method performs weighted regressions with an intercept term, allowing the assessment of pleiotropy among the IVs, with the slope being the estimate of the causal effect (25). The MR-Egger regression model is the only straight line in the axes that does not automatically pass through the origin. When the proportion of invalid IVs is as high as half and the precision of the estimates varies considerably between IVs, the WME approach still provides credible effect estimates (26). Simple mode, which is a less accurate method than weighted mode, uses causal effect estimates from individual SNP to form clusters. Weighted mode method uses the same process but assigns weights to each SNP. While they may not be as robust as the IVW and WME methods in detecting causal effects, they do outperform MR-Egger regression (27). The MR-PRESSO method was utilized to identify and remove outliers (SNPs with potential pleiotropy) and to estimate the corrected results (16).

Subsequently, we conducted a meticulous sensitivity analysis to identify and correct possible violations of the MR assumptions. Firstly, the IVW and MR-Egger methods were used to perform separate Cochran’s Q-tests and Rücker’s Q-tests to assess whether there was heterogeneity among IVs. A significance level of P < 0.05 was utilized to indicate potential heterogeneity, which was further visualized through a funnel plot. Notably, the presence of heterogeneity did not affect the results of the random-effects IVW estimation when the overall heterogeneity was balanced (24). The Steiger directionality test can be used to determine direction by calculating the proportion of variance explained by the IVs on the pair of exposure and outcome (28). To assess the validity of the overall direction of causality, we conducted an MR-Steiger directionality test for the validity of the hypothesis that exposure leads to outcomes (28). It is important to note that Steiger analyses can only provide preliminary evidence to verify directionality, not to establish causality. Additionally, the MR Egger intercept was employed for the horizontal polynomiality test, where a Y-intercept of zero would indicate the absence of any horizontal polynomial effect. Horizontal polynomiality is also detected by the MR-PRESSO global test, which estimates global heterogeneity to detect polynomiality (14, 16). A p-value of below 0.05 indicates the presence of pleiotropy in the selected IVs. Finally, for a comprehensive evaluation of the robustness of MR results, leave-one-out (LOO) analyses were employed, which evaluate whether the exclusion of one SNP had an impact on the overall effect of the remaining SNPs. It should be noted that the IVW method yields a statistically significant result while other complementary approaches do not exhibit statistical significance, and no evidence of pleiotropy was observed, one can deem it a favorable result, provided that the beta values of the other complementary approaches exhibit concordant directionality (29).

In this study, we corrected the p-values using the Bonferroni calibration method. As a consequence, we considered a Bonferroni-corrected threshold of p < 0.01 (p < 0.05/five exposures/one outcome) to be statistically significant as evidence that a causal relationship exists. It was considered suggestive evidence of causality if the P-value was between 0.05 and 0.01. The statistical analysis procedure described above was performed using the TwoSampleMR package (version 0.5.6) as well as the MRPRESSO package (version 1.0) within the R software (version 4.2.1). The TwoSampleMR code is freely available on this website under the following link: https://mrcieu.github.io/TwoSampleMR.

### 2.5 Reverse Mendelian randomization analysis

In the reverse MR analysis, we set the significance threshold of the p-value for IVs in psychiatric disorders more leniently to less than 5 × 10^-6^ to obtain sufficient SNPs. However, AN and AXD still failed to find usable SNPs in AS-associated GWAS despite a lower significance threshold of p < 5 × 10^-6^ after a series of stringent screenings. Therefore, for AN and AXD we relaxed the threshold of the p-value of exposure-associated IVs in the screening condition to < 5 × 10^-5^ and the linkage disequilibrium [LD] r ^2^ to 0.01. The settings above were previously also used in many MR studies of psychiatric disorders (30, 31). The causal effect of psychiatric disorders on AS is currently analyzed using the MR analysis methods described above except for phenotype “AN”, which applies only to the WR method because only one SNP is available. The valid SNPs obtained after rigorous screening for estimation of the MR causal effects of mental disorders on AS are listed in **Supplementary Table** **S4**.

## 3 Results

### 3.1 Research overview

**Figure 2:**
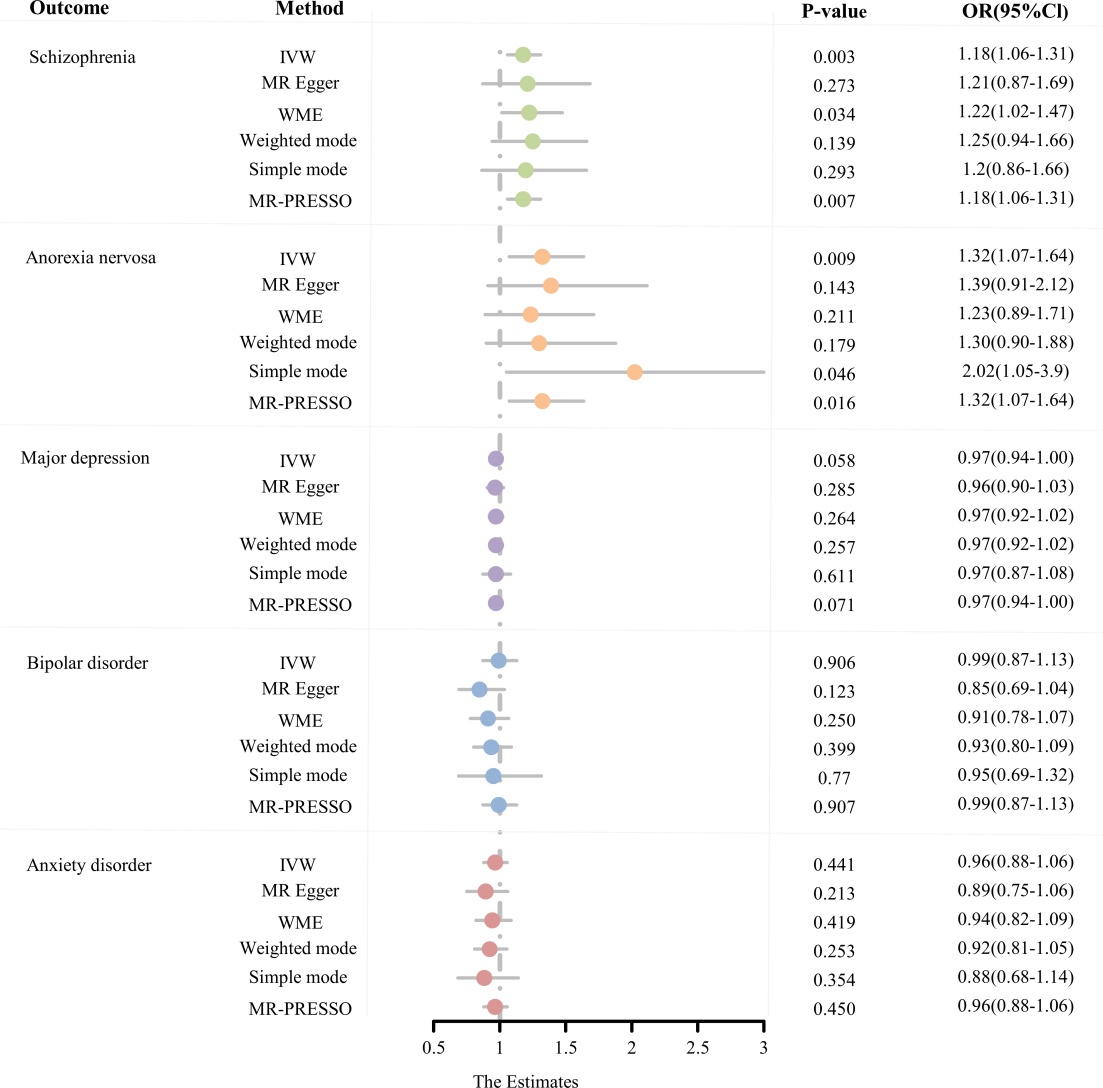
The MR estimates derived from different MR methods for the causal effect of exposure (ankylosing spondylitis) on outcome (five psychiatric disorders) are represented in a forest plot. (MR, Mendelian randomization; OR, odds ratio; CI, confidence interval; IVW, inverse variance weighted; WME, weighted median estimate; MR-PRESSO, Mendelian random pleiotropy residual sum and outliers).

The number of SNPs for AS as an exposure factor for each psychiatric phenotype ranged from 21 to 24 after a rigorous screening procedure. Based on the F-statistics, all of them were greater than 10, indicating that there is no bias caused by the utilization of feeble IVs (**Supplementary Table S3**). MR estimates obtained from the different methods are presented in **Figure 2**, refer to **Supplementary Table S5** for more details.

#### 3.1.1 MR analysis for the causal effects of AS on SCZ and AN

**Figure 3:**
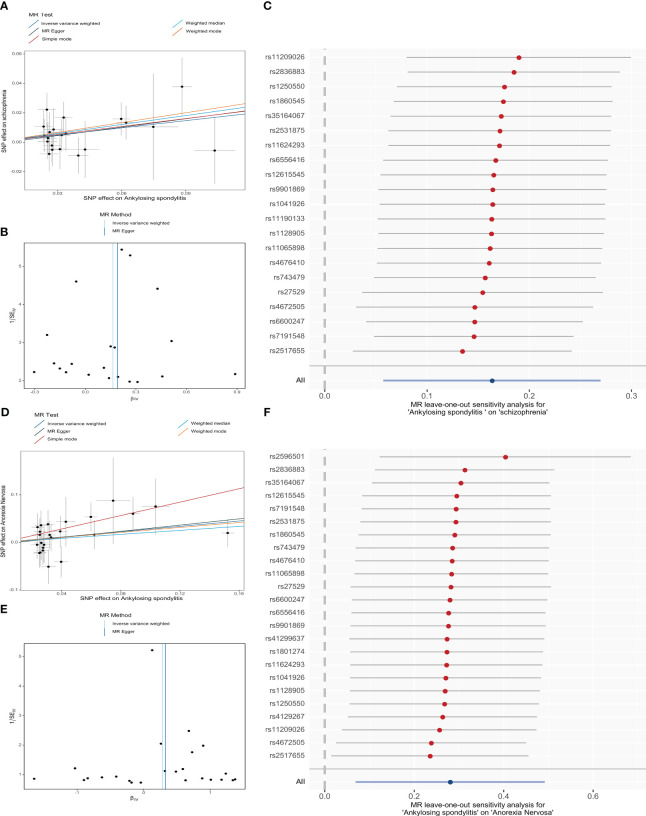
MR analysis of genetically predicted associations of ankylosing spondylitis on schizophrenia **(A-C)** and anorexia nervosa **(D-F)** visualized by Scatter plots (the slopes of the lines represent causal associations), Funnel plots, and Leave-one-out forest plot, respectively. (SNP, single-nucleotide polymorphism).

From the MR estimates we observe a causal effect of AS on SCZ and AN. To be specific, the causal-effect estimates of the IVW model suggested that AS significantly enhanced the risk of SCZ (OR = 1.18 95% CI 1.06-1.31, P = 2.58 × 10^-3^). Moreover, analogous causality estimates were also obtained from MR-PRESSO (OR = 1.18 95% CI 1.06-1.31, P = 6.85 × 10^-3^) and WME (OR = 1.22 95% CI 1.02-1.47, P = 3.36 × 10^-2^) models in the AS on SCZ MR analysis. Of these, the MR estimates were reliable for the MR-PRESSO method, with p = 6.85 × 10^-3^ remaining significant at the Bonferroni-corrected significance threshold (p < 0.05/5 = 0.01). Results from the WME method (P = 3.36 × 10^-2^) offered suggestive evidence of a potentially cause-effect association. Furthermore, as shown in **Figures 3A**, **3D**, the overall trend is consistent even though the estimates from the other complementary MR methods do not achieve statistical significance. In the MR analysis of AS on AN, the causal estimates of the IVW model indicated that AS was significantly associated with an elevated risk of developing AN (OR = 1.32, 95% CI: 1.07-1.64, P = 9.43 × 10^-3^). Consistent causality estimates were also obtained from MR-PRESSO (OR = 1.32, 95% CI 1.07-1.64, P = 1.62 × 10^-2^) and the simple mode (OR = 2.02, 95% CI 1.05-3.90, P = 1.43 × 10^-2^) approaches. As the p-values were above the calibrated p-value, both provided suggestive causality. As well, other complementary MR methods offer consistent estimates in the direction of causality (**Figures 3A, D**).

Then, sensitivity analyses demonstrated that our MR conclusions were robust (**Supplementary Table S7**>). Cochran’s Q-test and Rücker’s Q-test showed that there was no heterogeneity in IVs (p > 0.05). The MR-PRESSO model did not detect SNPs with potential pleiotropy (p > 0.05). Additionally, the MR-PRESSO global test and the MR-Egger intercept results revealed that there exists no horizontal pleiotropy in the selected IVs (p > 0.05). Asymmetry of SNP estimation accuracy scales in IVW and MR Egger methods is not shown in funnel plots (**Figures 3B, E**). LOO analysis reflects the robustness of conclusions independent of individual SNPs (**Figures 3C, F**). The Steiger directionality test showed that AS exhibits a positive directional in causality with SCZ and AN (p < 0.05).

#### 3.1.2 MR analysis for the causal effects of MDD on BIP and AXD

Based on Bonferroni correction thresholds, the IVW methods of MR estimation showed that genetically predicted AS did not have a statistically significant effect on the risk of MDD (OR = 0.97, 95% CI: 0.94-1.00, P = 5.77 × 10^-2^), BIP (OR = 0.99, 95% CI: 0.87-1.13, P = 9.06 × 10^-1^), and AXD (OR = 0.96, 95% CI: 0.88-1.06, P = 0.41 × 10^-1^). Results from other MR analysis methods similarly support this conclusion (**Figure 2**, **Supplementary Table S5**). Furthermore, no outliers were observed for MR-PRESSO based on the results of sensitivity analyses (all p > 0.05), and MR estimates were not affected by heterogeneity and horizontal pleiotropy (all p > 0.05). LOO analysis of overall effects shows no influence from a single SNP (**Supplementary Figure S1**). The results of the Steiger directionality assessment support the validity of the causal relationship’s orientation (**Supplementary Table S7**). In conclusion, we did not find a significant causal relationship between AS and MDD, BIP, or AXD.

### 3.2 Reverse MR analysis

**Figure 4:**
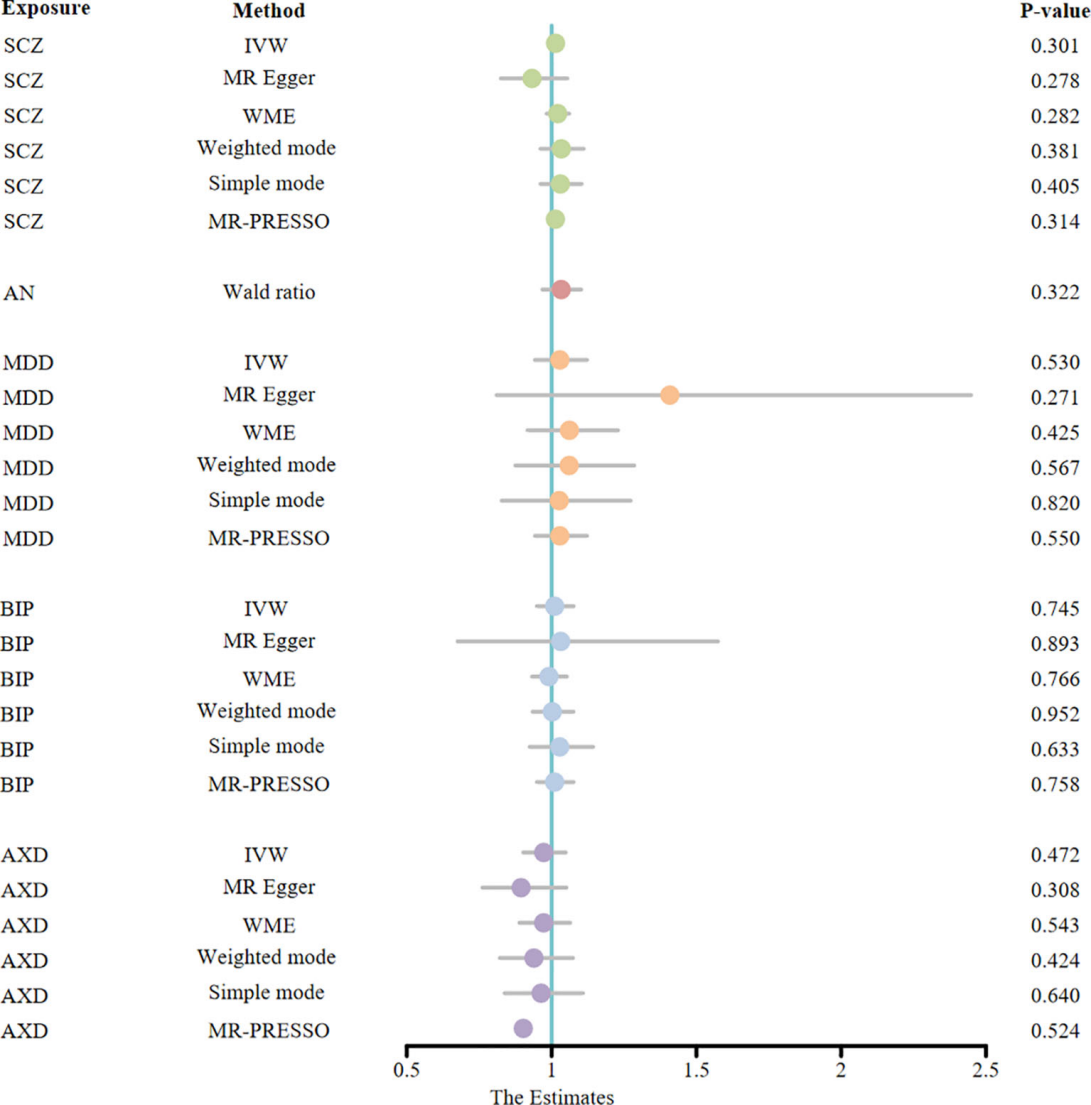
The MR estimates derived from different MR methods for the causal effect of exposure (five psychiatric disorders) on outcome (ankylosing spondylitis) are represented in a forest plot. (MR, Mendelian randomization; OR, odds ratio; CI, confidence interval; IVW, inverse variance weighted; WME, weighted median estimate; MR-PRESSO, Mendelian random pleiotropy residual sum and outliers; SCZ, schizophrenia; AN, anorexia nervosa; MDD, major depression disorder; BIP, bipolar disorder; AXD, Anxiety disorder).

To explore the reverse causality of psychiatric disorders on AS, we performed a reverse MR analysis. In this study, we obtained IVs ranging from 1-20, all of which had F-statistics > 10, suggesting that bias due to weak IVs could be neglected (**Supplementary Table S4**). The results of the MR estimation of the IVW method show that SCZ (OR = 1.01, 95% CI: 0.99 - 1.04, P = 0.30), AN (OR = 1.03, 95% CI: 0.97-1.10, P = 0.32), MDD (OR = 1.03, 95% CI: 0.94-1.12, P = 0.53), BIP (OR = 1.01, 95% CI: 0.94-1.08, P = 0.75), and AXD (OR = 0.97, 95% CI: 0.90-1.08, P = 0.76) did not have a statistically significant effect on the risk of AS. As depicted in **Figure 4**, none of the MR causal effect estimates for any of the complementary methods were statistically significant in terms of the causal association of mental illness on AS (all p > 0.01) (**Supplementary Table S6**). Hence, the hypothesis of reverse causation between mental illness and AS fails to hold. Importantly, our sensitivity analysis, employing MR-PRESSO, failed to detect any SNPs exhibiting pleiotropic effects (all p > 0.05). Additionally, we observed neither heterogeneity nor horizontal pleiotropy among the genetic instruments (all p > 0.05) (**Supplementary Table S8**, **Supplementary Figure S2**). The funnel plot exhibits a roughly symmetrical distribution (**Supplementary Figure S2**). Notably, in the LOO analysis, no specific SNP unduly influenced the MR estimates (**Supplementary Figure S2**). Moreover, the findings from the Steiger directionality test affirm the veracity of the causal directionality. It is noteworthy that the p-values for MDD and AXD did not attain significance (both p > 0.05). However, the fundamental causal direction remains true (**Supplementary Table** **S8**). We must acknowledge that, in the reverse MR study investigating AN as the exposure and AS as the outcome, we obtained only one usable SNP despite relaxing the criteria for IV selection. Consequently, we did not use MR methods such as IVW and MR Egger to analyze heterogeneity and horizontal pleiotropy.

## 4 Discussions

While potential causal relationships between AS and psychiatric disorders have been demonstrated in previous studies, as yet, there have been no conclusions drawn regarding the causal association between AS and psychiatric disorders (especially BIP and SCZ). It is possible that the interference of external confounding factors has not been excluded in most studies, leading to some conflicting discoveries. Researchers frequently employ statistical techniques to address confounders in observational studies by incorporating known and measured confounders into regression models. Nonetheless, when confounders remain unobserved or unknown, or when their number is considerable, regression methods may fail to produce unbiased estimates of the genuine connection between an exposure and outcome. As a result, the interpretation of findings can potentially introduce biases (32). For instance, an observational study in Sweden on the relationship between rheumatic diseases and mental disorders, in which many unmeasured or unknown potential confounders, such as environmental risk factors and substance use, were included in the study’s limitations (8). The presence of confounding variables can disrupt the estimation of exposure-outcome correlations and undermine the precision of causal determinations about study outcomes. Consequently, the demonstration of a direct causal relationship is rendered unattainable. Moreover, it should be noted that some observational studies, especially cross-sectional studies, may not be able to definitively identify temporal associations between AS and psychiatric disorders. To overcome this constraint and elucidate causation, longitudinal analyses are of utmost importance. Essentially, the prospect of reverse causality must be taken into account, whereby the occurrence of psychiatric disorders may plausibly impact the genesis of AS (11, 33).

In this study, we performed a two-sample MR analysis using publicly available GWAS summary data for the first time to assess causal associations between genetically predicted AS and five psychiatric disorders by bidirectional assessment. SNPs were utilized as instrumental variables that were not subject to confounders or reverse causality (34). The present findings suggest a potential causal effect of AS on SCZ and AN. More specifically, AS was significantly associated with an increasing risk of developing SCZ and AN. However, there were no psychiatric traits identified that showed reverse causality with AS, which included SCZ, AN, BIP, AXD, and MDD. This study provides genetic evidence for a cause-and-effect correlation between AS and psychiatric disorders. Which plays a crucial role in the development of psychiatric disorders.

So far, the exact mechanisms linking AS and mental disorders have not been fully understand. The following assumptions deserve to be considered. In recent findings, immune dysregulation has been shown to be one of the primary risk factors in the pathogenesis of psychiatric disorders (35–39). Genetic studies have shown that genes involved in immune modulation (major histocompatibility complex loci) are significantly correlated with SCZ (40). Thus, there may be a shared risk immunogenetic link between AS and SCZ. Furthermore, cytokines function in the periphery and brain as key signaling molecules of the immune system. Cytokines produced by the AS autoimmune process reach the brain through a variety of mechanisms, including brain barrier leakage, active transport, and bound specific cytokine receptors, which play a crucial role in mediating crosstalk between the immune system and the brain, and thus may be a potential contributor to psychiatric disorders (41, 42). A previous study showed higher levels of interleukin (IL)-1β and IL-6 production in patients with AS compared to normal subjects (43). Solmi et al. found that an association of elevated circulating concentrations of the cytokines tumor necrosis factor (TNF)-α, IL-1β, and IL-6 with AN (44). In addition, several meta-analyses have found that cytokine abnormalities such as (altered levels of inflammatory factors such as IL-1β, IL6, IL10, and TNF-α) are associated with SCZ (45). Cytokines such as TNF-α have been shown to have anorexic effects on the hypothalamus and peripheral anorexic effects *via* leptin (41, 46, 47). The SCZ cytokine model indicates that there is increased inflammation to be found in the brains of SCZ patients (42). Proinflammatory cytokines induce increased production of kynurenine through activation of the tryptophan metabolic pathway, which is involved in the regulation of the glutamate and serotonin systems, and kynurenine is converted to the N-methyl-D-aspartic acid receptor (NMDA) antagonist kynurenine in astrocytes. NMDA receptor hypofunction is associated with the pathophysiology of SCZ (48). As well, there are other biological events that can be triggered by cytokine activity, such as the hypothalamic-pituitary-adrenal activation axis (49), increasing oxidative stress (50, 51), diminished hippocampal volume, and promotion of dopaminergic sensitization (42). Hyperactivation of the HPA axis can be reliably observed in AN and SCZ (42, 52). Besides, imbalances in the gut microbiota may also be a potential mechanism for AS and AN. One pathophysiological model of AN may stem from altered signaling between the microbe-brain axis that regulates feeding behavior. The model suggests that the caseinolytic protease B homologue (ClpB) produced by Escherichia coli is a mimic of the α-melanocyte-stimulating hormone, a neuropeptide expressed by neurons in the arcuate nucleus of the hypothalamus, which produces a feeling of satiety (53). It was found that levels of ClpB were higher in patients with AN compared to healthy controls (54). Studies have shown that the onset and development of AS are associated with changes in the composition of microorganisms (55). Ciccia et al. found a significantly increased abundance of Escherichia coli and Prevotella in the terminal ileum of AS individuals (56). This may increase levels of ClpB and may increase the risk of developing AN. However, the precise mechanisms by which AS leads to SCZ and AN still need to be further explored and more thoroughly researched.

In this analysis, we employed the IVW method as the primary statistical approach, which demonstrated superior statistical efficacy in comparison to alternative methods like MR-Egger. MR-Egger, due to its limited statistical efficacy, exhibited wider confidence intervals and insignificant P values when compared to IVW. Thus, IVW is commonly employed as the principal criterion for identifying potentially significant outcomes. To ensure the reliability of the IVW estimates, this study implemented additional MR methods and conducted sensitivity analyses. Additionally, it is crucial to ensure the absence of horizontal pleiotropy, wherein IVs utilized in MR analyses solely impact the target outcome through the intended exposure. Horizontal pleiotropy manifests when genetic variations influence traits unrelated to the pathway of interest but still affect the target outcome, or when the variations directly impact the target outcome (57). The presence of horizontal pleiotropy introduces distortions to the MR test, resulting in erroneous estimations of causality, diminished statistical power, and the potential for false-positive causal associations. Should horizontal pleiotropy be detected, it is advisable to refer to the results obtained from MR-Egger, as this method adjusts IVW analyses by accounting for imbalanced or directed horizontal pleiotropic effects across all SNPs (22). Consistent with the conventions of most MR analyses, our study adheres to the requisite of consistent β-direction for all MR methods (58).

In our study, we have several strengths: Firstly, it is a two-sample MR study with large-scale GWAS statistics, which minimizes the likelihood of reverse causality and potential confounding in observational studies. This considerably enhances the reliability and persuasiveness of our study. Secondly, summary statistics were obtained from samples of European ancestry. It effectively reduces bias due to population stratification. Third, due to the fact that the data were collected from different organizations, there was little overlap between exposures and outcomes in the sample. Fourthly, multi-sensitivity analysis results demonstrated that there was the non-existence of potential pleiotropy in the instrumental variables indicating the robustness of our results. In the fifth place, the prevalence of AN in individuals with AS has not been investigated epidemiologically to our knowledge. The causal effect of AS on AN obtained in this study may guide future epidemiology. Lastly, we also performed a reverse MR analysis to determine that there is no reverse causality of psychiatric disorders on AS. There are inevitably some limitations to our study. The first concern is the possibility of bias in the GWAS summary statistics, such as coding errors, misclassification of cases, etc. Next, stratified analyses based on factors such as age and gender were not possible owing to the limitations of the GWAS summary data. Thirdly, currently, there have been fewer GWAS studies on AS, the reliability of the findings could be further validated by obtaining a larger exposure sample and extracting SNPs more closely associated with AS. Furthermore, in our MR analysis of reverse causality, we relaxed the significance threshold setting for p-values for IVs associated with mental disorders, which means that the proportion of variance explained by some valid IVs is relatively small. This may have contributed to the nonsignificant difference between the proportion of variance explained by IVs for MDD and AXD and the proportion of variance explained for AS in the Steiger directionality test. An insufficient number of IVs were available for AN. It was not possible to perform a sensitivity analysis to verify the robustness of the results. Moreover, given that we used GWAS summary data from individuals of European ancestry, caution should be exercised in generalizing the findings to other populations due to potential inter-ethnic genetic variations. It is imperative that data from other populations are analyzed and compared to make the results more trustworthy. Finally, while the MR-PRESSO examination or MR-Egger intercept evaluation did not discern significant pleiotropy and confounding elements, the potentiality of these factors cannot be entirely dismissed. Such is a pervasive predicament encountered in the realm of MR analyses.

## 5 Conclusions

In summary, the evidence presented in the two-way MR studies suggests that there may be a causal correlation between genetically predicted AS and the development of SCZ and AN. Our investigation provides valuable insights into intervening in psychiatric disorders (SCZ and AN) among individuals with AS. Further research in the future is imperative to unravel the intrinsic mechanisms that underlie the association between ankylosing spondylitis and SCZ and AN.

## Data availability statement

The original contributions presented in the study are included in the article/**Supplementary Material**. Further inquiries can be directed to the corresponding author.

## Ethics statement

The studies involving humans were approved by the present analysis was based on publicly available summary-level data, which did not need additional approval. The studies were conducted in accordance with the local legislation and institutional requirements. Written informed consent for participation in this study was provided by the participants’ legal guardians/next of kin.

## Author contributions

HZ: Writing – original draft, Writing – review & editing. MML: Writing – review & editing.
