# Supplementary material for: Ankylosing spondylitis and psychiatric disorders in European population: a Mendelian randomization study
Source: Front Immunol. 2023 Oct 26;14:1277959. doi: 10.3389/fimmu.2023.1277959 (PMC10637577; doi:10.3389/fimmu.2023.1277959)
Supplement: Supplementary file 2 [file DataSheet_1.docx]

Supplementary Material

# Supplementary Figures

**Supplementary Figure S1**. Scatter plots, Funnel plots and Leave-one-out analysis of genetically predicted associations of ankylosing spondylitis with major depressive disorder(A-C) and anxiety disorder (D-F), and bipolar disorder (G-I).

**Supplementary Figure S2**. Scatter plots, Funnel plots and Leave-one-out analysis of genetically predicted associations of schizophrenia (A-C), major depressive disorder (D-F), bipolar disorder (G-I), and anxiety disorder (J-L) with ankylosing spondylitis.


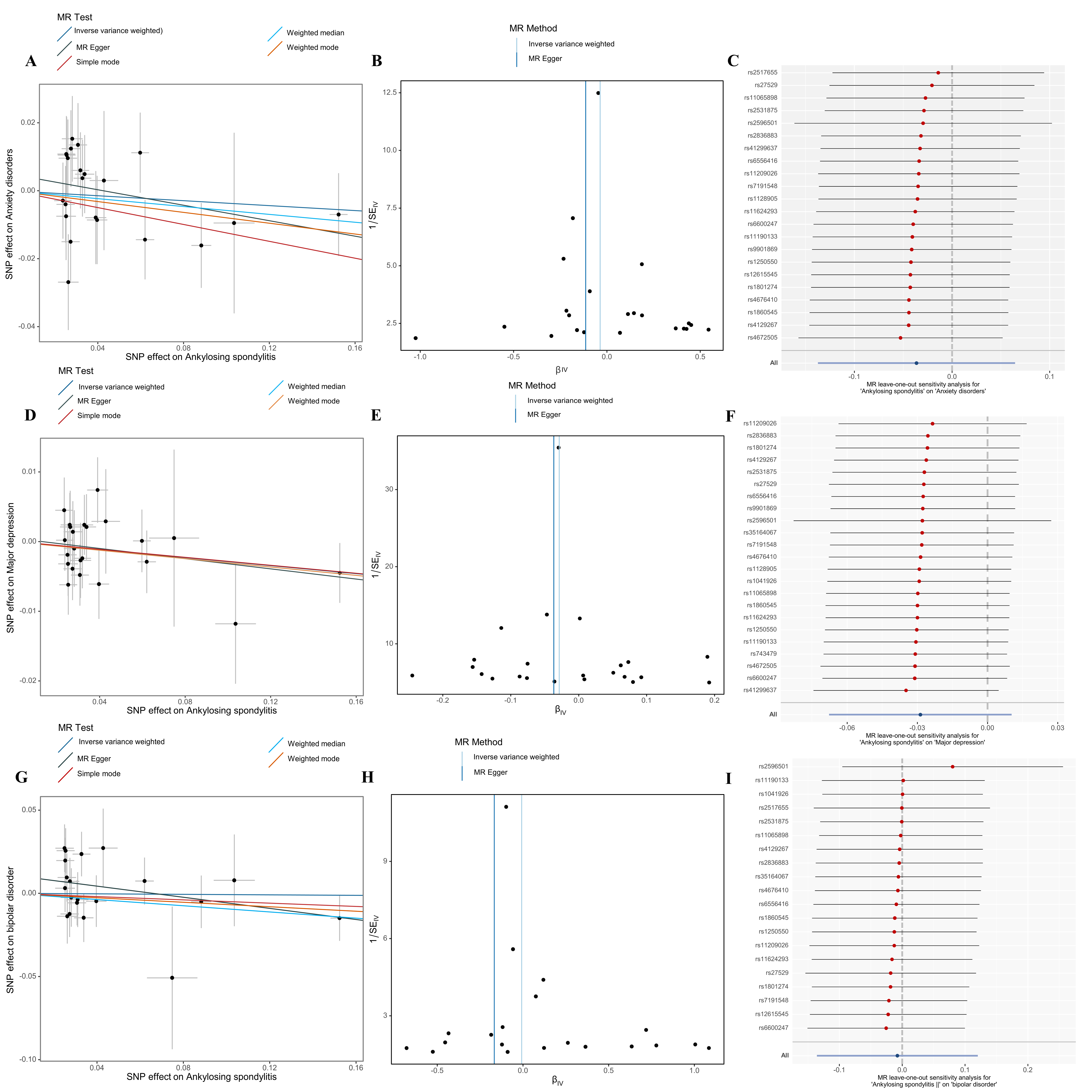


**Supplementary Figure S1**. Scatter plots, Funnel plots and Leave-one-out analysis of genetically predicted associations of ankylosing spondylitis with major depressive disorder(A-C) and anxiety disorder (D-F), and bipolar disorder (G-I). (Note: Scatter plots showing robust genetic associations of psychiatric disorders with ankylosing spondylitis. The slope of the line represents the causal association, and each method has a different line.Funnal plot to assess heterogeneity. Less precise estimates (lower values on y-axis) 'funnel' in as they increase in precision. Leave-one-out sensitivity analyses were conducted to determine whether a given association was disproportionately affected by a single SNP. Each red point in the forest plot indicates an MR analysis that excludes that specific SNP .)


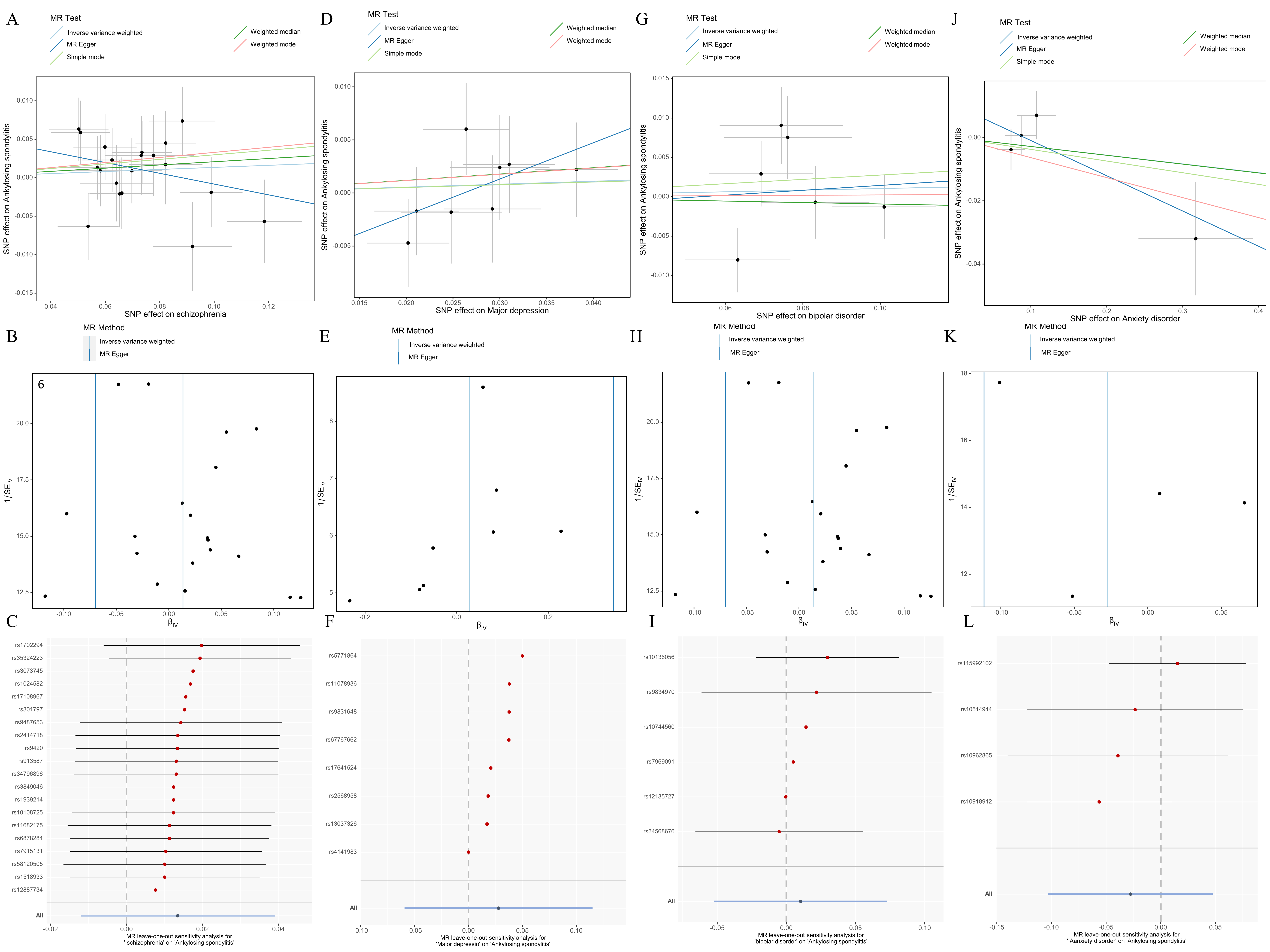


**Supplementary Figure 2**. Scatter plots, Funnel plots and Leave-one-out analysis of associations of genetically predicted schizophrenia (A-C), major depressive disorder (D-F), bipolar disorder (G-I), and anxiety disorder (J-L) with ankylosing spondylitis. (Note: Scatter plots showing robust genetic associations of psychiatric disorders with ankylosing spondylitis. The slope of the line represents the causal association, and each method has a different line.Funnal plot to assess heterogeneity. Less precise estimates (lower values on y-axis) 'funnel' in as they increase in precision. Leave-one-out sensitivity analyses were conducted to determine whether a given association was disproportionately affected by a single SNP. Each point in the forest plot indicates an MR analysis that excludes that specific SNP (using IVW).)
